# The study of the association between exercise motivation and cardiorespiratory fitness in young students: a systematic review and meta-analysis

**DOI:** 10.3389/fpsyg.2025.1566952

**Published:** 2025-03-21

**Authors:** Fangbing Zhou, Wenlei Wang, Yuyang Nie, Chunxue Shao, Wenxue Ma, Wentao Qiu, Guofeng Qu, Jinchao Gao, Cong Liu

**Affiliations:** ^1^College of Physical Education and Sports, Beijing Normal University, Beijing, China; ^2^College of Education for the Future, Beijing Normal University, Zhuhai, China; ^3^College of Physical Education, Northeast Normal University, Changchun, Jilin, China

**Keywords:** young students, physical activity, exercise motivation, physical fitness, intervention strategies

## Abstract

**Background:**

In recent years, many studies have shown that exercise motivation is essential for encouraging students to engage in physical activities. Cardiorespiratory function, which is closely related to cardiorespiratory fitness, plays a crucial supportive role in sports, and its level is usually reflected by cardiorespiratory fitness measurements. This study aims to explore the correlation between exercise motivation and cardiorespiratory fitness in young students, analyze the impact of exercise motivation on cardiorespiratory fitness, and investigate the role of cardiorespiratory fitness in the formation of exercise motivation.

**Methods:**

Following the PRISMA statement, a comprehensive literature search was carried out in six electronic databases from July 1, 2000, to December 1, 2024. The selected studies were strictly quality-assessed, and relevant data were extracted using a standardized form. Then, a meta-analysis was conducted with Stata18 software, along with heterogeneity testing and publication bias assessment.

**Results:**

After screening, 11 studies were included. Eight directly explored the correlation between cardiorespiratory fitness and exercise motivation, while the other four investigated the link between physical activity and exercise motivation, suggesting an association between cardiorespiratory fitness and exercise motivation during physical activity. Pearson correlation analysis (11 studies) and multiple regression analysis (7 studies) were used. By combining effect sizes with a random-effects model, the average correlation coefficient was 0.24 (*p* < 0.001). The average standardized coefficient of exercise motivation on promoting cardiorespiratory fitness was 0.16 (*p* < 0.001), and that of cardiorespiratory fitness on enhancing exercise motivation was 0.18 (*p* < 0.001).

**Discussion:**

The results show a significant positive correlation between exercise motivation and cardiorespiratory fitness in young students, with a moderate positive effect on each other. This provides a theoretical basis for improving young students’ cardiorespiratory fitness and exercise motivation. Future research could explore more effective assessment methods to better understand the underlying mechanisms.

## Introduction

In recent years, the issue of students’ physical fitness has garnered increasing attention from society. Particularly, the significant decline in cardiorespiratory fitness has raised high alert in the field of public health (Yin, 2017). As one of the important indicators of physical health, cardiorespiratory fitness is not only related to an individual’s daily activity capacity and quality of life but is also a key factor in preventing chronic diseases such as cardiovascular diseases (Wu and Wang, 2023). However, among the current student population, the level of cardiorespiratory fitness is generally low, which is directly related to the insufficient participation of students in sports. The World Health Organization (WHO) recommends that adolescents engage in at least 60 min of moderate to vigorous-intensity physical activity daily (Zhang et al., 2021; British Nutrition Foundation, 2004; National Association for Sport and Physical Education, 2005; Guo and Rao, 2021) to promote the improvement of cardiorespiratory fitness. However, actual surveys have found that the number of students who meet this physical activity standard is very few (Jing and Mu, 2020). This current situation indicates a significant lack of motivation for students to participate in sports, and exploring the underlying determinants is of great practical significance for improving students’ cardiorespiratory fitness.

In recent years, scholars have made positive progress in methods to promote exercise behavior. Numerous studies, based on various theoretical frameworks related to exercise motivation such as self-determination theory, planned behavior theory, and the health action process approach, have thoroughly investigated possible pathways to enhance students’ cardiorespiratory fitness. These theories provide a multi-dimensional perspective for understanding and enhancing students’ motivation to exercise. Research shows that motivation theories play a crucial role in explaining and promoting participation in physical activity. For example, self-determination theory emphasizes the importance of individuals’ autonomy, competence, and relatedness in exercise motivation, pointing out that when students feel they have the autonomy to choose to participate in sports, possess the ability to complete sports tasks (Liu et al., 2013; Zhu and Yin, 2017; Xue, 2010), and receive support and recognition from others during sports, their intrinsic motivation for exercise will be significantly enhanced. After the enhancement of exercise motivation, students are more likely to actively participate in sports, and regular exercise has a positive impact on cardiorespiratory function. The planned behavior theory analyzes students’ intention to participate in sports from three dimensions: attitude, subjective norm, and perceived behavioral control, suggesting that a positive attitude, social support, and a high level of perceived behavioral control are important prerequisites for students’ participation in sports (Polet et al., 2020; Wang et al., 2011). Active participation in sports is a key factor affecting cardiorespiratory function. The Health Action Process Approach focuses on the cognitive, emotional, and behavioral factors of individuals during the process of health behavior change, emphasizing the promotion of cardiorespiratory fitness through enhancing students’ health awareness, stimulating positive emotions, and cultivating good exercise habits. This theory provides theoretical support from the perspective of health behavior change for researching the relationship between the two, revealing the mechanism by which exercise motivation affects cardiorespiratory function through influencing the health behavior process (Zhang and Li, 2023; Wang and Zheng, 2020; Chang, 2013). These three theories, each with a different focus, elaborate on the relationship between individual internal psychological needs, external environment, individual cognition, the process of health behavior change, and exercise motivation. Exercise motivation, in turn, directly or indirectly influences sports participation and cardiorespiratory function, providing a comprehensive and in-depth theoretical foundation for researching the relationship between cardiorespiratory function and exercise motivation. This helps to reveal the complex intrinsic connections and mechanisms of action between the two Cardiorespiratory functions refer to the ability of the human heart to pump blood and the lungs to absorb oxygen, playing a fundamental and comprehensive key role in physical health (Franklin et al., 2022; Gordan et al., 2015). Higher cardiorespiratory fitness enables individuals to endure greater exercise intensity and longer duration during physical activity, reducing fatigue (Wang et al., 2022). When individuals can easily handle a certain level of physical activity during exercise, they are more likely to persist, and this persistence in exercise is closely related to exercise motivation, as continuous exercise experiences can influence an individual’s perception and attitude toward exercise (Diehl et al., 2018), thereby affecting subsequent motivation to exercise.

In the past academic research field, there has been a lack of comprehensive and systematic review studies on the correlation between young students’ sports motivation and cardiorespiratory endurance. Numerous literatures involving cardiorespiratory endurance and sports motivation merely mention the connection between the two, and the discussion is somewhat superficial. Some scholars have reviewed the correlation between physical activity and sports motivation; however, they have not conducted in-depth and targeted research on cardiorespiratory endurance, a key indicator. For example, in the study “Exercise, Physical Activity, and Self-Determination Theory: A Systematic Review” published by Teixeira et al. (2012) although a positive association between more autonomous forms of motivation and exercise behavior was clearly identified, the importance of cardiorespiratory endurance as a key indicator of physical health was overlooked. Some scholars have also conducted research from the perspective of cardiorespiratory endurance and physical literacy. For instance, Jiang et al. (2024) study systematically reviewed and summarized the relationship between cardiorespiratory endurance and physical literacy. The results showed a significant positive correlation between the sub-factors of physical literacy—motivation and confidence—and cardiorespiratory endurance. This finding further reinforces the notion of a close connection between cardiorespiratory endurance and motivation. However, it is worth noting that the study only revealed the correlation between the two and did not delve into the deeper mechanisms of their mutual influence. In addition, some research has focused on the relationship between physical activity and sports motivation in populations with health conditions. For example, Hutzler and Korsensky (2010) publication, “A Systematic Literature Review of the Motivation for Physical Activity in People with Intellectual Disabilities,” revealed the correlation between the motivation for individuals with intellectual disabilities to engage in sports, recreation, or health-related physical activities. Veldhuijzen Van Zanten et al. (2015) publication, “Perceived Barriers, Facilitators, and Benefits of Regular Physical Activity and Exercise in Patients with Rheumatoid Arthritis: A Literature Review,” demonstrated the relationship between physical activity barriers and lack of motivation in patients with rheumatoid arthritis. However, given the physiological and psychological differences between populations with health conditions and the general population, these research findings are not entirely applicable to the general population.

Although numerous studies have mentioned the association between cardiorespiratory endurance and sports motivation in their articles, there is a noticeable absence of meta-analyses specifically focusing on the in-depth relationship between cardiorespiratory endurance and sports motivation in general young students. Furthermore, previous meta-analyses have not comprehensively integrated multiple dimensions of sports motivation. In light of this, the present study aims to delve into this research gap, with the intention of providing new perspectives and insights for the field, and exploring the intrinsic psychological mechanisms underlying young students’ participation in physical activities from the perspective of psychological motivation. Through the review and analysis of relevant literature, the study seeks to reveal the impact mechanisms of different motivational factors on cardiorespiratory endurance, as well as the specific application and effectiveness of different theoretical frameworks in promoting students’ sports participation. The research objective is to provide scientific evidence for the development of targeted behavioral intervention strategies, offering customized recommendations for educational practice, with the aim of effectively enhancing young students’ cardiorespiratory endurance, thereby increasing their level of physical activity, ultimately improving the overall health status of adolescents, and laying a solid health foundation for their comprehensive development.

## Methods

### Literature search strategy

According to the standards specified in the “Preferred Reporting Items for Systematic Reviews and Meta-Analyses (PRISMA)” (Moher et al., 2009)statement, a structured electronic literature search was conducted. The search was performed by the first author on October 1, 2024, using computerized methods and included six electronic databases: Web of Science, PubMed, ProQuest, Scopus, ScienceDirect, and EBSCO. The search strategy was as follows: “[Title/Abstract] = (‘cardiorespiratory fitness’ OR ‘Aerobic fitness’ OR ‘cardiorespiratory fitness’ OR VO2max (Maximum Oxygen Uptake)) AND [Title/Abstract] = (‘Exercise motivation’ OR ‘Physical activity motivation’ OR ‘Fitness motivation’) AND [Title/Abstract] = (‘college students’ OR ‘university students’ OR ‘young’ OR ‘adolescents’).” Only peer-reviewed articles published in Chinese and English journals were included in this study. The search covered a time span from July 1, 2000, to December 1, 2024. We will exclude unpublished reports, conference abstracts, theses, and other forms of grey/gray literature from this systematic review.

### Inclusion and exclusion criteria

Inclusion criteria: ① Participants were middle school to university students with an average age of 11 years or older; ② Included assessment of exercise motivation; ③ Included assessment of cardiorespiratory fitness; ④ Quantitatively analyzed the relationship between exercise motivation and cardiorespiratory fitness; ⑤ Cross-sectional, longitudinal, or long-term study design; ⑥ Published in English.

Exclusion criteria: ① Studies focusing on special populations, such as those with cardiovascular disease, diabetes, etc.; ② Literature not published in peer-reviewed journals; ③ Study samples of less than 50 participants; ④ Did not provide data on the relationship between exercise motivation and cardiorespiratory fitness; ⑤ Review articles ⑥ non - English language.

After deduplicating the retrieved literature, two researchers independently screened the studies based on the inclusion and exclusion criteria. Initially, they conducted a preliminary screening of the article titles and abstracts to identify potentially relevant literature for full-text review. In addition, while including the studies confirmed through full-text review, we also meticulously checked the reference lists of the retrieved full-text articles and other systematic reviews to ensure that no additional eligible studies were missed. Finally, the included literature was determined through a joint check by the two researchers. If there were any discrepancies in the screening results, a third researcher was consulted to make the final decision.

### Data extraction

The data were extracted by two authors according to the inclusion criteria, and any disagreements were resolved through discussion. The extracted information included: (1) authors and year of publication; (2) study location; (3) type of study; (4) methods for assessing exercise motivation; (5) methods for assessing cardiorespiratory fitness; (6) statistical methods; (7) association indicators (correlation coefficients, standardized coefficients); (8) study results.

### Quality assessment of literature

The quality of the literature was assessed using the Quality Assessment Tool for Quantitative Studies (QualSyst) derived from criteria for primary research papers in different fields (Jing and Mu, 2020). QualSyst is a 14-item tool that allows for the evaluation of methodology and bias in both quantitative and qualitative studies with different research designs. Due to the observational design of the studies included in this review, items 5 (random allocation), 6 (investigator blinding), and 7 (participant blinding) were removed from QualSyst. Each item on the QualSyst was scored from 0 to 2 points to indicate whether the study met a criterion (0 = no, 1 = partial, 2 = yes). All scores were summed to create a total score. The total score was then converted into a percentage (i.e., the total study score divided by 22), and ratings were assigned as “excellent” (>80%), “good” (70–79%), “adequate” (55–69%), and “low” (<55%) (Liu et al., 2013; Zhu and Yin, 2017). Two researchers independently assessed the quality, and any discrepancies were resolved through discussion until a consensus was reached.

### Statistical analysis

Each included study provided data on the association between exercise motivation indicators and cardiorespiratory fitness in young students, as well as the sample size of the study. For longitudinal studies included the association data at baseline were selected for analysis; for studies that used multiple instruments for assessment, the association data were combined using meta-analysis. The transformed data were subjected to meta-analysis using Stata 18 software. Based on the results of the heterogeneity analysis, a fixed-effect model was adopted for the included literature when I^2^ was less than 50% and P was greater than 0.05, or a random-effects model when I^2^ was greater than or equal to 50% or P was less than 0.05. The level of heterogeneity was represented by the I^2^ index, categorized as low (I^2^ ≤ 25%), moderate (25% < I^2^ ≤ 50%), and high (I^2^ > 50%) (Liu et al., 2023; Higgins et al., 2003). Subgroup analysis was conducted in the presence of significant heterogeneity. To test for publication bias, funnel plots were calculated, and Egger’s test was performed, with the results of the analysis presented in forest plots.

## Results

### Study selection process

**Figure 1:**
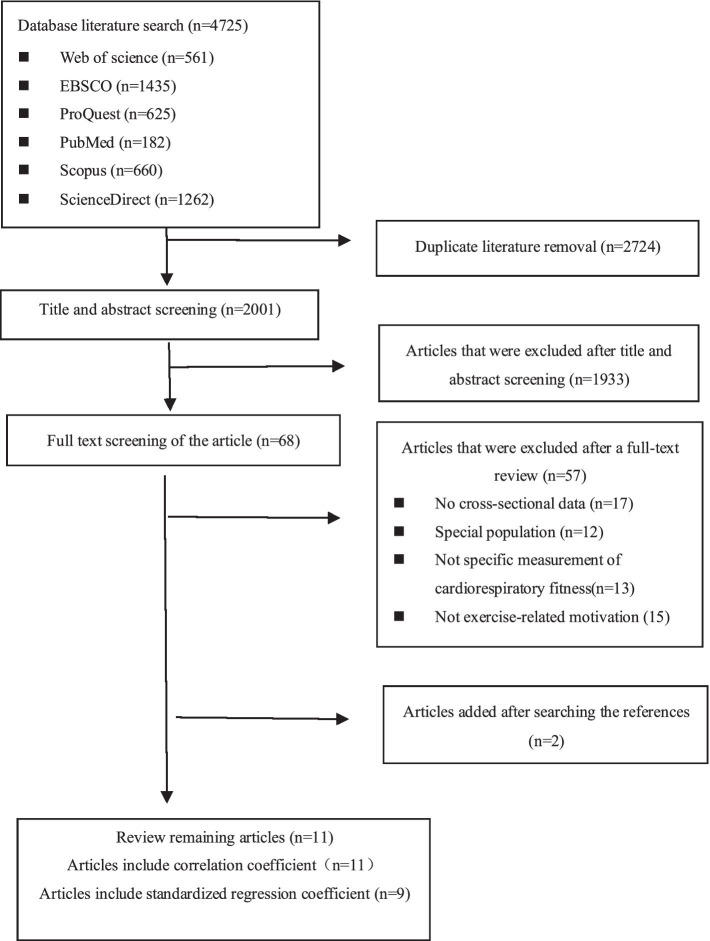
Flow chart of literature screening.

Figure 1 reports the study flow and reasons for excluding studies through the review process. A total of 4,725 potentially relevant articles were identified through database searches. After duplicates were removed, 2,001 unique articles entered the title and abstract screening, of which 1,933 were excluded. The full texts of the remaining 68 articles were reviewed according to the study selection criteria. Among these, 57 articles were excluded. The reasons for exclusion included: 17 articles lacked original data, 12 studies focused on special groups (such as patients with diseases, police officers, etc.), 13 studies did not specifically measure cardiorespiratory fitness, and 15 did not address exercise-related motivation. Ultimately, 11 articles met the inclusion criteria, spanning from July 2000 to October 2024.

### Basic characteristics of the included studies

Table 1 summarizes the basic information of the 11 included studies. All studies were published after the year 2000, with 8 cross-sectional studies and 3 longitudinal studies, all having relevant cross-sectional data. The study regions included countries such as China, the United Kingdom, Norway, and Canada, and the number of study participants ranged from 82 to 961 individuals. Among these studies, 11 included correlation analysis and correlation coefficients (r), and 8 studies employed multiple regression analysis and standardized coefficient (*β*) Detailed information is provided in Table 1.

**Table 1:** Summary of the included literature.

Number	Author, year	Study design	Country	Sample Size	Age (years)mean (SD) [range]	Percentage girls (%)	Instruments used (CRF)	Instruments used (EM)	Analysis	Association indicators	Conclusion
1	Kaj et al. (2015)	LS	Hung	961	12-20	70.09%	PACER	SCS	Multiple regression analyses	β = 0.249	Results suggest that boys and girls have different behavior characteristics and attitudes toward PE. Our study also suggests that different attitude components were associated with the aerobic capacity level in boys and girls
2	Shen and Xu (2008)	CS	CN	208	18–23 (20.1)	49.52%	800/1000	EMI-2	Bivariate correlations, Multiple regression analyses	r = 0.026 β = 0.081	Overall, a positive correlation was found between CF and self-efficacy for both men and women, he regression analyses. For men, CF was the only significant predictor for psychological motives when the contribution of BMI and self-efficacy was not significant. The model predicted 16% of the variance in psychological motives. However, for women, self-efficacy was the only significant
3	Zhang et al. (2022)	CS	CN	390	19.2 ± 1.2	51.13%	800/1000	PL	Bivariate correlations	r = 0.167	Furthermore, the PF domains of muscular strength and aerobic fitness were significantly and positively correlated with the PL attributes of confidence and physical competence in both men and women, while the PF domains of vital capacity and aerobic fitness were significantly and positively correlated with the PL attribute of motivation in men
4	Wilson et al. (2020)	LS	US	572	21.06 ± 1.07	39.6%	YMCA	SCS	Multiple regression analyses	β = 0.122	Findings suggest that, upon reflection, upperclassmen consider a lack of motivation as the greatest constraint to physical activity as a freshman.
5	Schneider (2018)	LS	US	142	11.04	52%	ramp-type progressive cycle-ergometer	BREQ	Multiple regression analyses	β = 0.081	Consistent with general trends, boys in this study were more active and had higher cardiorespiratory fitness as compared to girls. Boys also preferred higher-intensity exercise as compared to girls.
6	Power et al. (2011)	CS	US	82	12–14	51%	PACER	SCS	bivariate correlations	r = 0.102	intrinsic motivation was correlated with cardiovascular fitness, cardiovascular fitness was correlated with weight status
7	Li et al. (2022)	CS	CN	641	20.72 ± 1.41	50.55%	800/1000	MPAM-R	Bivariate correlations Multiple regression bootstrap	r = 0.336 β = 0.324	The physical exercise behavior of college students partially mediates the relationship between exercise motivation and cardiovascular fitness. Therefore, the educational concept of “Health First” should be promoted in college sports. Internal motivation of exercise can be transformed into external motivation to improve students’ exercise behavior and cardiovascular fitness through enhancing their cardiopulmonary capacity.
8	Riiser et al. (2014)	CS	NO	120	13.73	60.83%	PACER	BREQ-2	Multistage regression	β = 0.240	The physical exercise behavior of college students partially mediates the relationship between exercise motivation and cardiovascular fitness. Therefore, the educational concept of “Health First” should be promoted in college sports. Internal motivation of exercise can be transformed into external motivation to improve students’ exercise behavior and cardiovascular fitness through enhancing their cardiopulmonary capacity.
9	Gao et al. (2009)	CS	US	252	12-15	53.17%	PACER	SCS	Bivariate correlations, Multiple regression analyses	r = 0.226 β = 0.041	Correlation analysis yielded significantly positive relationships between ability beliefs and incentives. Regression results revealed that ability beliefs, importance, interest, and usefulness significantly predicted intention for future participation. Ability beliefs also emerged as significant predictors of PACER test scores.
10	Sibley et al. (2013)	CS	US	194	18-31	63.92%	PACER	MPAM-R	Bivariate correlations, Multiple regression analyses	r = 0.242 β = 0.081	MPAM-rand BreQ-2 subscales were regressed onto two fitness measures. MPAM-R, stronger competence and fitness motives predicted better fitness and stronger appearance motives predicted worse fitness。 For the BREQ2, higher intrinsic motivation predicted better aerobic fitness, and stronger, introjected regulation predicted higher body fat composition.
11	Cadenas-sanchez et al. (2021)	CS	ES	341	12–18	52.2%	PACER	AEQ-PE	Multiple regression analyses	β = 0.304	Boys presented with higher levels of cardiorespiratory fitness and motivation than girls. In addition, cardiorespiratory fitness was associated with higher motivation

CS, Cross sectional study; LS, Longitudinal l study; CRF, cardiorespiratory fitness; EM, Exercise motivation; SCS, Self-Created Scale; PL, Physical literacy; PACER, Physical Activity and Cardiovascular Endurance Run; BREQ, Behavioral Regulation in Exercise Questionnaire; EMI-2, Exercise Motivations Inventory version; MPAM, Motives for physical activities measure.

### Quality assessment of the included studies

The quality of the 11 included articles was assessed using the “Quality Assessment Tool for Primary Research Papers from Different Areas.” The scores of the 11 studies ranged from 68.2 to 100%, with an average score of 87.18%. Of these, 8 studies (72.73%) were rated as excellent quality, 2 study (18.18%) was rated as good quality, 1 study (9.09%) were rated as sufficient, as shown in Table 2.

**Table 2:** Results of the quality assessment of the studies included in the systematic review (*N* = 11).

Author and year	1. Research question	2. Studydesign	3. Subject and variable selection	4. Subject characteristics	8. Exposures and outcome	9. Sample size	10. Analytic methods	11. Estimate of variance	12. Confounding	13. Results in sufficient detail	14. Conclusions supporting results	Sums	Weights	Rank #
Kaj et al. (2015)	2	2	2	2	2	2	2	2	1	2	2	21	95.5%	Excellent
Gao et al. (2009)	2	2	2	2	2	2	2	2	2	2	2	22	100.0%	Excellent
Shen and Xu (2008)	2	1	2	1	2	2	2	1	1	1	2	17	77.3%	Good
Zhang et al. (2022)	2	1	2	2	2	2	2	2	2	2	2	21	95.5%	Excellent
Wilson et al. (2020)	2	2	1	2	2	2	2	1	1	2	2	19	86.4%	Excellent
Schneider (2018)	1	2	1	2	1	2	2	2	1	1	1	16	72.7%	Good
Power et al. (2011)	2	1	2	2	2	2	2	2	2	2	2	21	95.5%	Excellent
Li et al. (2022)	2	2	2	2	2	2	2	2	2	2	2	22	100.0%	Excellent
Riiser et al. (2014)	2	1	2	2	2	2	1	1	2	2	2	19	86.4%	Excellent
Cadenas-sanchez et al. (2021)	2	1	2	2	2	2	2	2	2	2	2	21	95.5%	Excellent
Sibley et al. (2013)	2	2	1	1	1	2	1	1	0	2	2	15	68.2%	Adequate

Each item was scored depending on to what degree the criterion was met: yes = 2 points, partial = 1 point, no = 0.; t study total sum score divided by the total possible score of 22; # A percentage score of > 80, 70–80%, 55–69, and < 55% was rated as “excellent,” “good,” adequate and “low,” respectively.

### Assessment tools for evaluating cardiorespiratory fitness in young students

This study included literature that employed four methods to assess the cardiorespiratory fitness of young students. Among them, six studies used the 20-meter shuttle run PACER (Progressive Aerobic Cardiovascular Endurance Run) (Kaj et al., 2015), a test method designed to evaluate an individual’s cardiorespiratory fitness. The test is conducted on a 20-meter straight track, and with each completed lap (i.e., 40 meters), the speed increases, typically controlled by specific music rhythms. Participants are required to keep up with the pace for as long as possible until they are unable to complete a lap within the specified time. Additionally, three studies adopted the 800-meter and 1,000-meter endurance tests from the Chinese National Physical Fitness Test (Shen and Xu, 2008). Furthermore, two studies used different methods, one of which employed the YMCA Sub maximal Cycle Ergometer test with two stage extrapolations while wearing a heart rate monitor. An estimate of maximal oxygen consumption was produced using the multistage model technique (Wilson et al., 2020). Another study used a ramp-type progressive bicycle ergometer exercise test to measure the maximum oxygen uptake (Schneider, 2018). These studies, utilizing various assessment tools, provide a multi-dimensional perspective on the evaluation of cardiorespiratory fitness in young students. The tools encompass both field and laboratory tests, as well as methods ranging from simple to complex, thereby ensuring the reliability and validity of the assessment results.

### Assessment tools for evaluating exercise motivation

In the literature included, seven main tools were used to assess the motivation of adolescents participating in sports. Among them, the “Behavioral Regulation in Exercise Questionnaire” (BREQ) was used in three studies. Based on self-determination theory, it assesses the types of motivation in individuals’ sports behavior, including intrinsic motivation and various forms of extrinsic motivation. Additionally, two studies employed the “Motives for Physical Activity Measure–Revised” (MPAM-R), a questionnaire designed to evaluate the intensity of motivation for individuals to engage in physical activities. This questionnaire, also based on self-determination theory, measures the motivation for participating in activities such as weightlifting, aerobics, or various team sports. Furthermore, four studies used self-created scales, including a questionnaire developed by the Hungarian School Sports Federation (HSSF) using standardized procedures to measure exercise motivation, and a questionnaire from Ryan and Connell (1989) in the United States to assess children’s motivation in the achievement and prosocial domains. These questionnaires evaluate four types of behavioral self-regulation derived from self-determination theory: external regulation, introjected regulation, identified regulation, and intrinsic motivation. Another questionnaire, adapted from previous research (Xiang et al., 2003, 2004) in the United States, measures exercise motivation subfactors (expectancy, importance, usefulness, interest) by posing relevant questions. Furthermore, one study used the “Exercise Motivations Inventory-2″ (EMI-2), a scale designed to assess the motivation of individuals to participate in sports. The EMI-2 is suitable for both athletes and non-athletes, and it reflects how an individual’s exercise motivation affects their level of sports participation and potential sports choices. Additionally, one study used the “Subjective Changes in Physical Activity Questionnaire,” where participants were asked to compare their current level of physical activity with their level during high school and their freshman year to evaluate how easy or difficult they found it to engage in sports during their freshman year. Challenges (i.e., limitations) faced during the freshman year, such as time management, lack of knowledge about how to exercise within the campus, having someone to exercise with, lack of motivation, and discomfort in using exercise facilities, were rated.

Finally, two studies used the “Physical Literacy Awareness Tool” (PLAT), which assesses an individual’s mastery of basic knowledge, skills, and attitudes toward physical activity. By improving physical literacy, more people can be encouraged to participate in sports activities, thereby promoting the healthy development of society. The other tool used was the “Achievement Motivation in Physical Education Questionnaire” (AMPEQ), which is designed to evaluate students’ achievement motivation in the field of physical education (Cadenas-sanchez et al., 2021)^.^

### Research progress

This study is the first to systematically and comprehensively review the relationship between exercise motivation and cardiorespiratory fitness in young students. After rigorous searching and screening, 11 high-quality literature articles were selected, and methods such as correlation analysis, multiple regression analysis, and Meta-analysis were employed for in-depth research. The results indicate a significant positive correlation between the two, with a bidirectional association and mutual influence. The study exploring the impact of exercise motivation on cardiorespiratory fitness shows that exercise motivation has a predictive effect on cardiorespiratory fitness. Conversely, when investigating the impact of cardiorespiratory fitness on exercise motivation, the results reveal that students with higher cardiorespiratory fitness also have higher exercise motivation. Additionally, factors such as gender, psychological cognition, and sports knowledge affect this relationship. Males generally have higher cardiorespiratory fitness and exercise motivation than females; stronger psychological cognitive abilities and beliefs are associated with higher exercise motivation and cardiorespiratory fitness; and individuals with a rich reserve of sports knowledge place greater importance on sports and have higher levels of cardiorespiratory health. In summary, the two factors mutually enhance each other, forming a virtuous cycle. This study provides theoretical support for promoting the development of cardiorespiratory fitness in young students, suggesting that intervention measures should aim to enhance exercise motivation and encourage student participation in sports.

### Meta-analysis

All 11 included studies provided data on the association between cardiorespiratory fitness and exercise motivation in young students. Among them, 6 studies directly provided correlation data between cardiorespiratory fitness and exercise motivation in young students. The remaining studies provided standardized beta coefficients, which were converted into correlation coefficients r using a formula for meta-analysis. The Pearson correlation coefficient r in the literature, combined with the Pearson coefficient and sample size, was converted into Fisher’s Z score, standard error (SE), and 95% confidence interval (CI) (Borenstein et al., 2011; Szczuka et al., 2021).A meta-analysis was conducted using a random-effects model for the correlation coefficient ® and the 95% confidence interval (CI). Before performing the calculations, the standardized coefficient *β* was converted to an r value using the formula r = β × 0.98 + 0.05*λ* (when-0.5 < β < 0, λ = −1;when 0 < β < 0.5, λ = 1) (Peterson and Brown, 2005) The results showed that the pooled effect size was 0.24, with a 95% confidence interval ranging from 0.19 to 0.30, indicating a significant overall effect among the studies. The heterogeneity test revealed a chi-square value of 35.50 with 11 degrees of freedom, and a *p*-value less than 0.0001, suggesting significant heterogeneity among the studies. The I-squared value was 72.45%, indicating that 72.45% of the variance in the effect size could be attributed to the heterogeneity between studies. In addition, the z-value for the Test of ES = 0 was 8.49, with a *p*-value less than 0.0001, further confirming the statistical significance of the study results.

**Figure 2:**
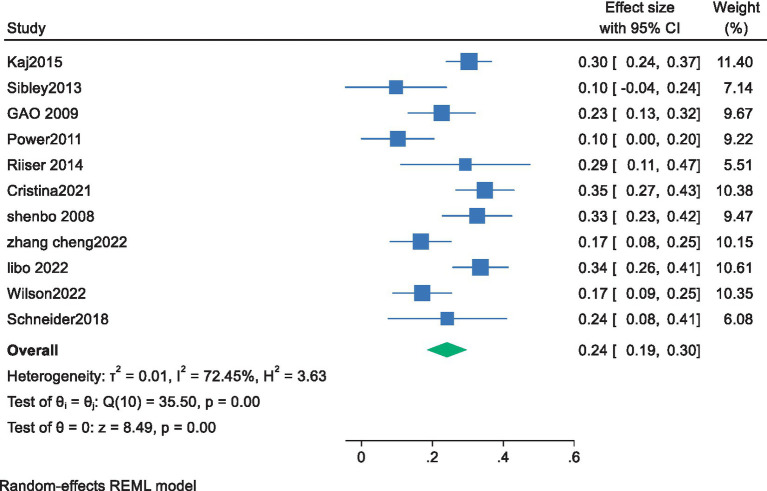
Forest plot of the association between cardiorespiratory fitness and exercise motivation among young.

The pooled Fisher’s z score and 95% CI were converted back to the correlation coefficient r, resulting in an r of 0.235 with a 95% CI. The lower limit of the 95% confidence interval was approximately 0.187, and the upper limit was approximately 0.291. This means that the analysis yielded an average correlation coefficient of 0.235 between cardiorespiratory fitness and exercise motivation in young students, with a 95% confidence interval ranging from 0.187 to 0.291 (Figure 2).

**Figure 3:**
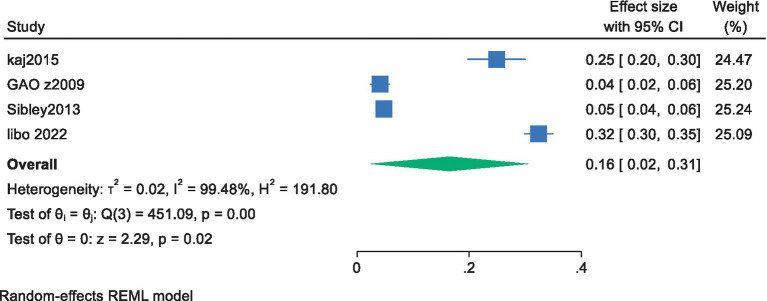
Forest plot of the effect of exercise motivation on cardiorespiratory fitness among young.

Of the 11 included studies, 4 conducted regression analyses with exercise motivation as the independent variable and cardiorespiratory fitness as the dependent variable, providing effect sizes (standardized coefficients) for the prediction of future cardiorespiratory fitness in adolescents based on exercise motivation. The 95% confidence intervals (CI) were calculated using the formula (CI = *β* ± 1.96 × SE) based on the standardized coefficient β and the standard error (SE). A meta-analysis was performed using a random-effects model for the standardized coefficient β and the 95% CI. The results showed a pooled effect size of 0.16, with a 95% confidence interval ranging from 0.02 to 0.31, indicating a significant overall effect among the studies. The heterogeneity test revealed a chi-square value of 451.09 with 3 degrees of freedom, and a *p*-value less than 0.0001, suggesting significant heterogeneity among the studies. The I-squared value was 99.48%, indicating that 99.48% of the variance in the effect size could be attributed to heterogeneity among the studies. Additionally, the z-value for the test of ES = 0 was 2.29, with a *p*-value less than 0.05, further confirming the statistical significance of the results (Figure 3). The overall results suggest that exercise motivation has a moderate predictive effect on the future level of cardiorespiratory fitness in young students, with a standardized coefficient of 0.16. This means that for everyone standard deviation unit increase in exercise motivation, the probability of an increase in the future level of cardiorespiratory fitness in young students averages an increase of 0.16 standard deviation units.

**Figure 4:**
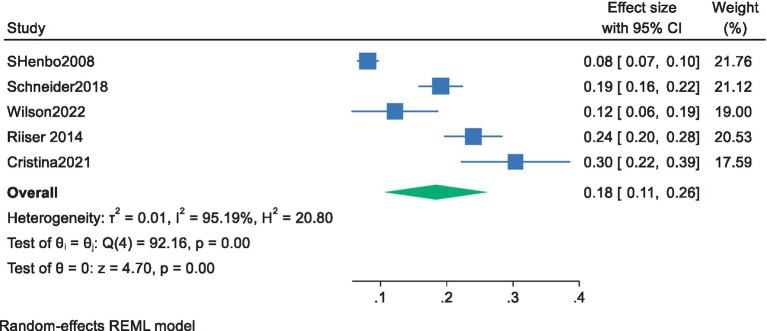
Forest plot of the effect of cardiorespiratory fitness on exercise motivation among young.

Of the 11 included studies, 5 conducted regression analyses with cardiorespiratory fitness as the independent variable and exercise motivation as the dependent variable, providing effect sizes (standardized coefficients) for the impact of cardiorespiratory fitness on adolescents’ exercise motivation. The 95% confidence intervals were calculated using the formula (confidence interval = *β* ± 1.96 × SE) based on the standardized coefficient β and the standard error SE. A meta-analysis was performed using a random-effects model for the standardized coefficient β and the 95% CI. The results showed a pooled effect size of 0.18, with a 95% confidence interval ranging from 0.11 to 0.26, indicating a significant overall effect among the studies. The heterogeneity test revealed a chi-square value of 92.16 with 4 degrees of freedom, and a *p*-value less than 0.0001, suggesting significant heterogeneity among the studies. The I-squared value was 95.19%, indicating that 95.19% of the variance in the effect sizes could be attributed to heterogeneity among the studies. Additionally, the z-value for the Test of ES = 0 was 4.70, with a *p*-value less than 0.0001, further confirming the statistical significance of the results (Figure 4). The overall results showed that cardiorespiratory fitness has a moderate effect on the exercise motivation of young students, with a standardized coefficient of 0.18. This means that for everyone standard deviation unit increase in cardiorespiratory fitness, the exercise motivation of young students increases by an average of 0.18 standard deviation units.

### Subgroup analysis

**Figure 5:**
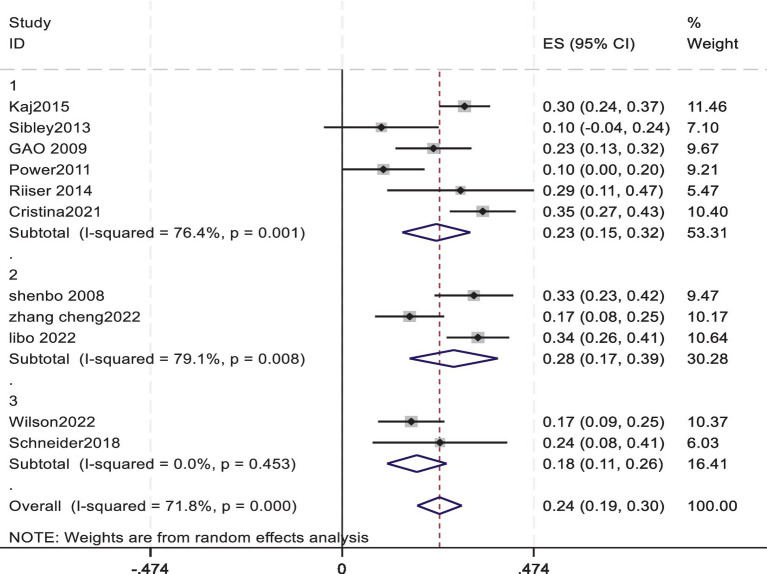
Forest plot of Subgroup analysis.

A subgroup analysis was conducted based on different cardiorespiratory fitness assessment tools. Specifically, the data were divided into three subgroups using the 20-meter shuttle run PACER, the 800/1000-meter run, and other assessment tools. The subgroup analysis was performed based on the cardiorespiratory fitness assessment tools (PACER, 7 studies; 800/1000-meter run, 3 studies; other tools such as the YMCA heart rate monitor and ramp-type progressive bicycle ergometer, 2 studies) to examine the association between adolescent cardiorespiratory fitness and exercise motivation. The results showed that the pooled effect size for PACER was 0.23, with a 95% confidence interval of 0.15 to 0.32; for the 800/1000-meter run, the pooled effect size was 0.28, with a 95% confidence interval of 0.17 to 0.39; and for other assessment tools, the pooled effect size was 0.18, with a 95% confidence interval of 0.11 to 0.26. The *p*-values for all three subgroups were less than 0.05, indicating a significant overall effect among the studies (Figure 5). This suggests that there is a significant association between adolescent cardiorespiratory fitness and exercise motivation overall, regardless of the different cardiorespiratory fitness assessment tools used, meaning that cardiorespiratory fitness has a notable impact on exercise motivation. The heterogeneity test results showed that the I-squared value for the subgroup using PACER was 76.4%, and for the subgroup using the 800/1000-meter run, it was 79.1%, both indicating a high degree of heterogeneity among the studies within these two subgroups. This heterogeneity may be due to significant differences in study subjects, research methods, interventions, and other factors across the studies. Meanwhile, the subgroup using other assessment tools had an I-squared value of 0.0%, suggesting low heterogeneity and more consistent results within this subgroup. However, this subgroup only included two studies with a small sample size, so its reliability needs further validation. The differences in results obtained from different assessment tools suggest that caution is needed when selecting cardiorespiratory fitness assessment tools. The choice should be made based on a comprehensive consideration of the research objectives, study subjects, and other factors to select the most appropriate assessment tool. Additionally, further research is needed to develop more accurate and effective cardiorespiratory fitness assessment tools to better investigate the relationship between cardiorespiratory fitness and exercise motivation.

**Figure 6:**
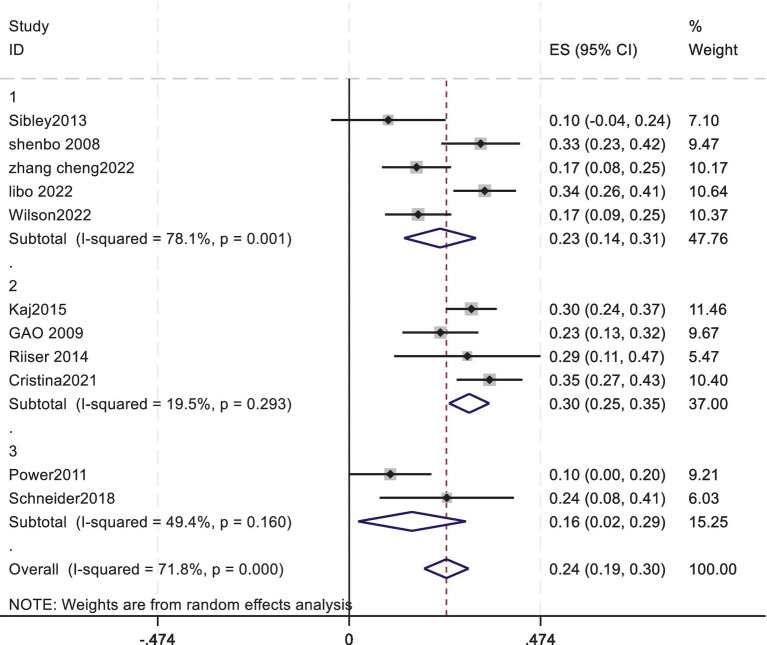
Forest plot of Subgroup analysis.

Based on the above-mentioned research data, subgroup analyses were carried out to further explore the sources of heterogeneity. According to the educational stages, the included studies were divided into three different subgroups: primary school, middle school, and university, with 2 studies in the primary - school subgroup, 4 studies in the middle - school subgroup, and 5 studies in the university subgroup, respectively. The results of the subgroup analyses revealed a significant positive correlation between cardio - respiratory fitness and exercise motivation across different educational levels. Specifically, the pooled effect size of the primary - school subgroup was 0.16, with a 95% confidence interval ranging from 0.02 to 0.29. The pooled effect size of the middle - school subgroup was 0.30, with a 95% confidence interval from 0.25 to 0.35. The pooled effect size of the university subgroup was 0.23, with a 95% confidence interval from 0.14 to 0.31. The *p* - values of all three subgroups were less than 0.05, indicating a significant overall effect statistically, thus demonstrating that cardio - respiratory fitness has a significant impact on exercise motivation. Regarding the heterogeneity assessment, the I^2^ statistic of the primary - school subgroup was 49.4% and that of the middle - school subgroup was 19.5%. Both indicated low heterogeneity. This indicates that the correlation between cardiopulmonary endurance and exercise motivation plays a significant role in both primary and secondary school stages, demonstrating a certain degree of consistency in the research findings. In contrast, the university subgroup showed high heterogeneity, with an I^2^ value of 78.1%. This might be attributed to the wider age range within this group. This observation emphasizes the need for greater caution when interpreting the research findings of the university subgroup and the consideration of other variables that may affect the results (Figure 6).

**Figure 7:**
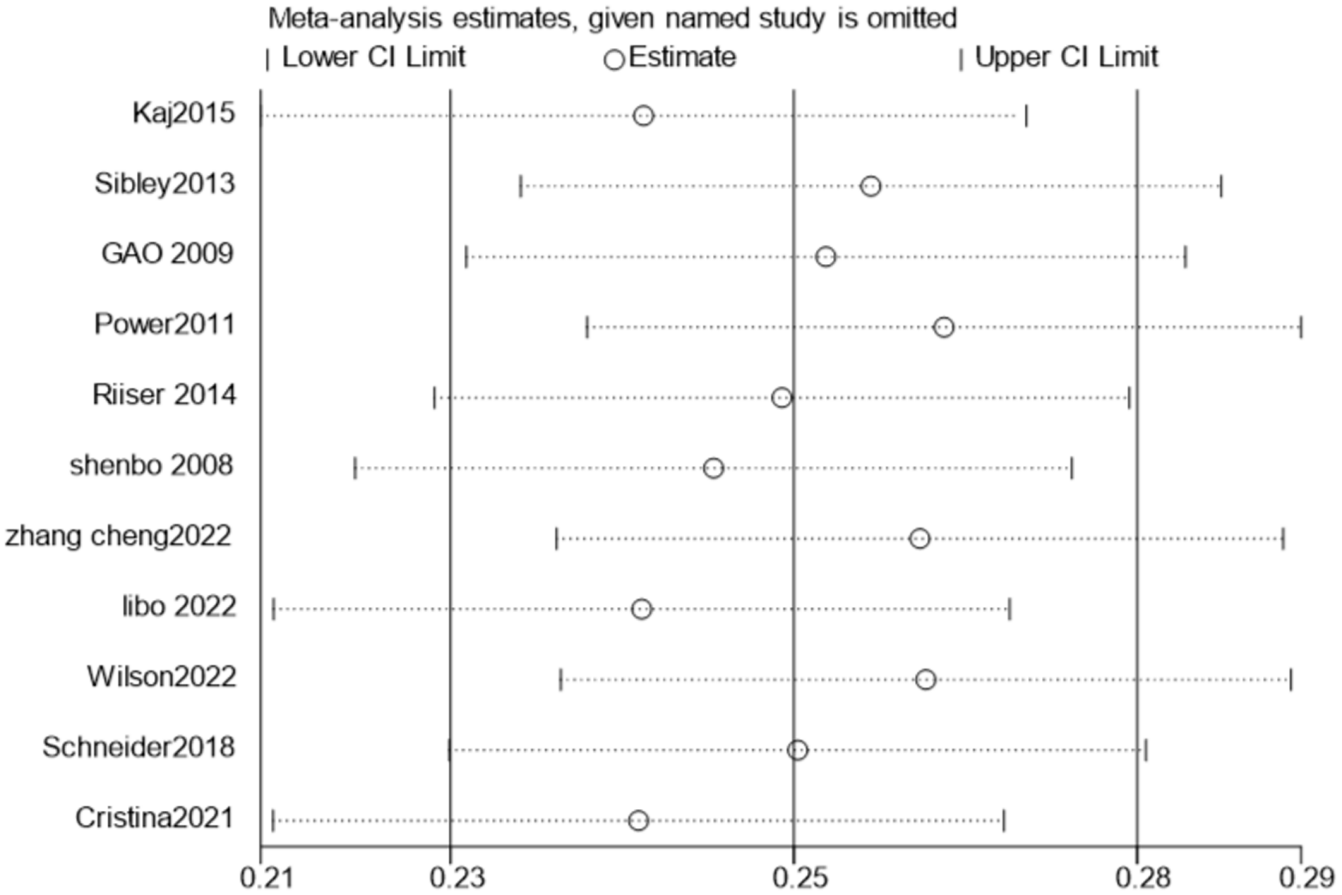
The results of the sensitivity analysis for the included studies.

Overall, although there are differences in the degree of association between cardio - respiratory fitness and exercise motivation among students at different educational stages, a significant positive correlation was consistently observed. This finding highlights the importance of improving students’ cardio - respiratory fitness to enhance their exercise motivation. In addition, the results of this study emphasize the necessity of considering heterogeneity when choosing research tools and interpreting research conclusions. Finally, we conducted a sensitivity analysis on this outcome, indicating the stability of the results (Figure 7).

### Publication bias assessment and sensitivity analysis

**Figure 8:**
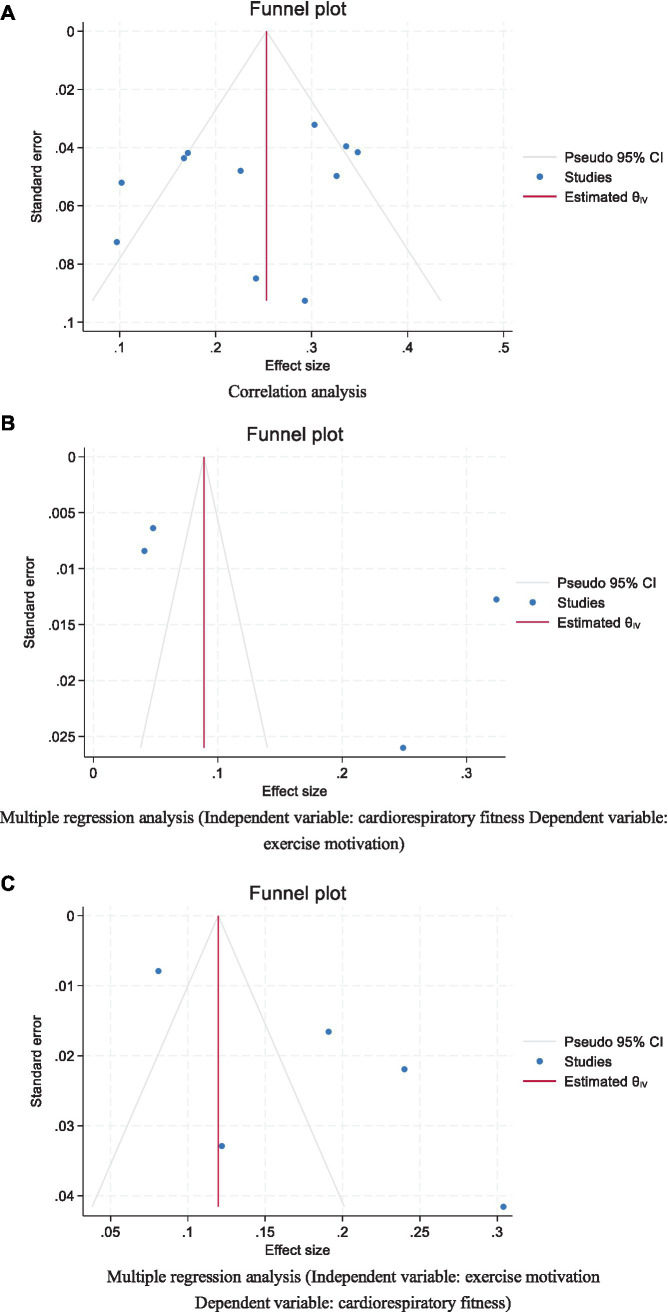
Funnel plot analyzing the association between cardiorespiratory fitness and exercise motivation among young. **(A)** Correlation analysis. **(B)** Multiple regression analysis (Independent variable: cardiorespiratory fitness Dependent variable: exercise motivation). **(C)** Multiple regression analysis (Independent variable: exercise motivation Dependent variable: cardiorespiratory fitness).

The funnel plot exhibits symmetry, indicating that the effect sizes are uniformly distributed around the overall effect size on both sides. The blue dots are symmetrically distributed within the grey lines, and the majority of them fall within the grey lines, suggesting minimal publication bias. In conjunction with the Egger’s regression test, which yielded a *p*-value of 0.317 for the intercept across various data types, we conclude that there is no evidence of publication bias in the statistical correlation data (r) (Figure 8A).

The funnel plot displayed exhibits symmetry, yet the distribution of blue dots beyond the confines of the grey lines suggests the potential presence of publication bias. In light of the Egger’s regression test outcomes, which returned a *p*-value of 0.285 for the intercept across various data types, it is inferred that an acceptable magnitude of bias is present within the standardized coefficient beta (Figure 8B).

The funnel plot illustrated demonstrates a certain degree of symmetry, indicating that the effect sizes are uniformly distributed around the estimated overall effect size. However, the presence of several blue dots outside the grey lines may suggest the potential for publication bias. Considering the results of Egger’s regression test, which yielded a *p*-value of 0.093 for the intercept across various data types, we infer that there is an acceptable level of bias in the standardized coefficient beta. This finding still helps to strengthen our confidence in the overall integrity and reliability of the data under consideration (Figure 8C).

The funnel plots and Egger’s test were employed to assess the publication bias of the included studies. By examining the symmetry of the funnel plots, in conjunction with the *p*-values of the interception coefficients for various data types from Egger’s test (0.317, 0.287, and 0.093), we conclude that there is no evidence of publication bias in the statistical correlation data r. Furthermore, regarding the standardized coefficient *β*, there is insufficient evidence to indicate the presence of small-study effects or publication bias (Table 3).

**Table 3:** Results of publication bias test.

Date type	*P*-value of slope	*P*-value of bias	Is there publication bias
Correlation data r	*p* = 0.005	*p* = 0.317	No
Standardized β	*p* = 0.626	*p* = 0.285	No
Standardized β	*p* = 0.391	*p* = 0.093	No

The sensitivity analysis indicated that there was no substantial change in the positive association when each study was omitted one by one (Figure 7).

## Discussion

This study comprehensively and systematically reviews the relationship between exercise motivation and cardiorespiratory fitness among young students for the first time. Through systematic database searches, 11 research articles were successfully selected, covering cross - sectional and longitudinal studies, all of which are of high quality. During the research process, correlation analysis and multiple regression analysis were mainly used. After standardizing similar correlation data, a Meta - analysis was carried out. The results show that there is a significant statistical association between exercise motivation and cardiorespiratory fitness among young students. In addition, the Meta - analysis of multiple regression reveals that the influence between cardiorespiratory endurance and exercise motivation is bidirectional. Individuals with strong cardiorespiratory endurance also have stronger exercise motivation, and the level of exercise motivation can predict the future level of cardiorespiratory endurance.

The results of the Meta - analysis of correlation coefficients indicate that there is a moderate positive impact between cardiorespiratory endurance and physical activity among young students, with a significant positive correlation (the average correlation coefficient r = 0.24, *p* < 0.001). This means that students with stronger cardiorespiratory endurance typically have higher exercise motivation. A systematic review on the sports motivation of Chinese college students (Deng et al., 2023) shows that the influencing factors include the sports level. The study reveals that individuals with a lower sports level also have weaker exercise motivation. In addition, another Meta - analysis study on the impact of motivational physical activity interventions on the aerobic fitness (CRF) of healthy adults further confirms the association between exercise motivation and cardiorespiratory endurance (Chase and Conn, 2013). Motivational physical activity (PA) interventions have a significant effect on improving the aerobic fitness of healthy adults, indicating an association between stronger exercise motivation and stronger cardiorespiratory endurance. In the study by Galan-lopez et al. (2022), the researchers explored the potential links between cardiorespiratory fitness (CRF) and different subfactors of exercise motivation. The results show that when CRF levels increase, boys scores on exercise motivation subfactors such as revitalization and enjoyment, competition, strength and endurance, and challenge also increase. For female participants, when CRF levels rise, there is an increase in scores on exercise motivation subfactors like recovery and enjoyment, strength and endurance, and challenge. This indicates that individuals with higher cardiorespiratory fitness feel more relaxed during exercise and are less prone to fatigue, thereby being more willing to participate in physical activities, which in turn strengthens their exercise motivation. There is a mutual influence and interaction between exercise motivation and cardiorespiratory fitness. Another study explored the correlation between exercise motivation and cardiorespiratory fitness from a different perspective. Raghuveer et al. (2020) research suggests that the results of cardiorespiratory fitness tests are also influenced by motivation levels. In summary, there is a moderate correlation between exercise motivation and cardiorespiratory fitness.

To further explore the correlation between cardiorespiratory fitness and exercise motivation in adolescents, this study conducted a subgroup analysis of the relevant data. Specifically, we divided the analysis into three categories based on different cardiorespiratory fitness assessment tools: the 20-meter Pacer test, the 800/1000-meter run test, and other assessment tools. The analysis results showed that in all three subgroups using these assessment tools, there was a significant positive correlation between adolescents’ cardiorespiratory fitness and exercise motivation. This finding indicates that the selected assessment tools have a certain degree of validity in measuring the association between cardiorespiratory fitness and exercise motivation.

In addition, the results of the Meta-analysis of multivariate/regression data further confirm this point. In the Meta-analysis with exercise motivation as the independent variable and cardiorespiratory fitness as the dependent variable, the average standardized coefficient *β* was 0.16 (*p* < 0.001). The study indicates that students with stronger exercise motivation typically have higher levels of cardiorespiratory fitness; conversely, students with weaker exercise motivation, due to a lack of sufficient motivation, have lower enthusiasm for participating in sports, resulting in relatively weaker cardiorespiratory fitness. Therefore, exercise motivation has a predictive effect on cardiorespiratory fitness. This conclusion is highly consistent with the research findings presented in the article “Research on the Relationship between Physical Activity Constraints among College Freshmen and Current Health, Behavior, and Outcomes among Upperclassmen” by Wilson et al. (2020). In this study, differences in freshmen’s physical activity constraints, current physical activity levels, predicted aerobic fitness, and obesity based on subjective changes in physical activity were explored through one-way ANOVA of subjective physical activity changes. The research found that compared to their high school years, students who reported a decrease in physical activity indicated that they were more limited by motivation during their freshman year, and the difficulty in exercising significantly increased. This indicates that a decrease in exercise motivation makes students less willing to participate in physical activity, which in turn leads to a gradual decline in cardiorespiratory fitness levels. Thus, it can be seen that exercise motivation is an important factor in predicting future cardiorespiratory fitness.

However, in terms of the impact of sub-factors of exercise motivation on future cardiorespiratory health, Kaj et al. (2015) further confirmed the predictive role of exercise motivation in cardiorespiratory endurance in their longitudinal study, while also finding significant gender differences. Using multiple regression analysis, the study found that various sub-factors of exercise motivation, including health orientation in physical education (C1), avoidance of failure (C2), success orientation (C3), attitude toward physical activity (C4), and cooperation and social experience (C5), were all significantly related to cardiorespiratory health (*p* < 0.05). Specifically, for boys, the statistically significant predictors included C1, C2, C3, and C4, while for girls, they were C2 and C4. This indicates that there are significant differences between male and female students in terms of motivational factors predicting future cardiorespiratory health.

Additionally, in the Meta-analysis where cardiorespiratory fitness was the independent variable and exercise motivation was the dependent variable, the effect size was 0.18 (*p* < 0.001). The research results indicate that students with stronger cardiorespiratory fitness typically have stronger exercise motivation, further clarifying the association between cardiorespiratory fitness and exercise motivation. The study by Riiser et al. (2014) also supports this viewpoint; when conducting a regression analysis of self-determined motivation on cardiorespiratory fitness, a statistically significant relationship was found, with a regression coefficient B = 2.08, 95% CI [0.53–3.63], *p* < 0.01, suggesting that healthier adolescents tend to have stronger exercise motivation.

In addition, we conducted a publication bias test on the included literature. Through the funnel plot and Eggers test, we did not find publication bias in the correlation data r and standardized coefficient *β*, which enhances the reliability of the results of this study. These findings provide an important empirical basis for further exploring the relationship between exercise motivation and cardiorespiratory fitness and offer references for future research directions and methodologies.

Overall, the relationship between cardiorespiratory fitness and exercise motivation in young students manifests as a positive feedback loop. Students with stronger cardiorespiratory fitness typically have higher exercise motivation, and those with stronger exercise motivation are likely to develop greater cardiorespiratory fitness in the future, with the two factors mutually reinforcing each other to create a virtuous cycle. This bidirectional relationship suggests that in interventions aimed at enhancing cardiorespiratory fitness in young students, it is important not only to focus on increasing exercise motivation but also to encourage active participation in physical activities to strengthen cardiorespiratory fitness. These studies provide important theoretical support for the development of cardiorespiratory fitness in young students.

When delving into other factors that affect the correlation between cardiorespiratory fitness and exercise motivation, it becomes evident that the relationship between the two is influenced by numerous external factors. Among these, gender, psychological cognition, and educational level are particularly crucial. In the comprehensive analysis of the 11 studies, 6 of them pointed out that males generally have higher levels of cardiorespiratory fitness and exercise motivation than females. For instance, Schneider (2018) study showed that, consistent with the overall trend, boys in the study were more active than girls, had higher levels of cardiorespiratory fitness, and were more inclined to participate in high-intensity sports. Furthermore, Wilson et al. (2020) research indicated that females were less active than males, and among females, those with lower levels of aerobic fitness were more limited by comfort factors, facing greater difficulties during physical activity. Thus, gender differences play a significant role in the association between exercise motivation and cardiorespiratory fitness.

Secondly, from the perspective of psychological cognition, there is a significant association between an individual’s beliefs in their cognitive abilities and both exercise motivation and cardiorespiratory fitness. Gao et al. (2009) study provides strong empirical support for this. Through correlation analysis, the study clearly indicated a significant positive relationship between ability beliefs and motivation. Further regression analysis results showed that factors such as ability beliefs, importance, interest, and usefulness significantly predict an individual’s intention to participate in related activities in the future. Particularly noteworthy is that ability beliefs are a key predictor of scores on the PACER 20-meter shuttle run test. This fully demonstrates that the stronger an individual’s beliefs in their cognitive abilities, the more intense their exercise motivation and the higher their level of cardiorespiratory fitness.

In addition, the study by Shen et al. (2009) found that the reserve of sports knowledge also plays a significant role in the relationship between exercise motivation and cardiorespiratory fitness. The research indicates that individuals with a richer reserve of sports knowledge tend to place greater importance on sports and are inclined to engage in physical activities to promote their health. Specifically, the study showed that students in the mastery-oriented group differed significantly from those in other groups in terms of effort in classroom learning, knowledge acquisition, and cardiorespiratory health levels. Compared to students in the performance-oriented rich group and the low motivation group, the mastery-oriented students not only possessed more knowledge but also had a more proactive attitude toward physical education (as evidenced by interest and perceived autonomy) and demonstrated higher levels of cardiorespiratory health. Furthermore, some data were not included in the meta-analysis due to a lack of necessary quantitative data, but their perspectives are of great value. For instance, Failde-Garrido et al. (2022) divided students into physically active and inactive groups based on their cardiorespiratory fitness levels and physical activity questionnaire scores. They used the BREQ questionnaire to assess the exercise motivation of the active and inactive groups, and the results showed that the active group had significantly higher exercise motivation than the inactive group. Additionally, the study concluded that educational level had an impact on physical activity, with higher-educated individuals engaging in more sports activities and exercising more (Corder et al., 2009), while those with lower educational levels had a higher prevalence of lack of physical activity and overweight (Devaux and Sassi, 2013).

Although this study provides important insights into the correlation between exercise motivation and cardiorespiratory fitness in young students, it also has several limitations. First, the predominantly cross - sectional design of this study implies a lack of exploration of the causal relationship between cardiopulmonary endurance and exercise motivation. Second, subgroup analyses conducted to assess school levels, measurement tools, and confounding factors help reduce heterogeneity, indicating their importance as sources of heterogeneity. However, there may be other unmeasured factors that influence this association, such as insufficient sample representativeness, large differences in sample size, few longitudinal studies, inconsistent assessment tools, lack of consideration for other potential factors and their interactions, high heterogeneity in the Meta-analysis, potential publication bias, challenges in interpreting results and applying them in practice, and a limited scope of research. To address these issues, in future experimental research, it is essential to deeply explore the influencing mechanisms of gender, psychological awareness, sports knowledge, and educational levels, conduct long - term tracking research using diverse methods, and evaluate the intervention effects, so as to optimize the strategies for improving exercise motivation and cardiorespiratory endurance. In the subsequent teaching practice, personalized exercise programs should be designed by taking gender differences into account. It is necessary to focus on enhancing psychological awareness and popularizing sports knowledge. Attention should also be paid to differences in educational levels, and comprehensive intervention measures should be adopted.

## Conclusion

This study has confirmed a significant positive correlation between exercise motivation and cardiorespiratory fitness in young students, with the positive influence of exercise motivation on cardiorespiratory fitness being moderate, and conversely, cardiorespiratory fitness also having a positive effect on exercise motivation. This finding provides a theoretical basis for enhancing cardiorespiratory fitness and strengthening exercise motivation in young students. Future research could also explore more effective assessment methods to gain a more comprehensive and in-depth understanding of the association mechanism between exercise motivation and cardiorespiratory fitness.

## Data Availability

The original contributions presented in the study are included in the article/supplementary material, further inquiries can be directed to the corresponding authors.
